# The Dedalo Project, a Community-based Prevention Program for the Promotion of Healthy Behaviors in Adult Population: Model Description and Target Population Assessment

**DOI:** 10.1007/s10935-022-00693-0

**Published:** 2022-07-14

**Authors:** Sara Bortoluzzi, Alessandro Coppo, Daniela Alessi, Stefano Parovina, Sara Napoletano, Irene Ammazzagatti, Chiara Airoldi, Angelica Zibetti, Chiara Aleni, Silvia Caristia, Fabrizio Faggiano, DEDALO Coordinating Group, Alessandro Coppo, Daniela Alessi, Antonella Barale, Maria Luisa Berti, Claudia Taliano, Marilena Mento, Fabrizio Faggiano

**Affiliations:** 1 School of Hygiene and Preventive Medicine, Department of Translational Medicine, Università degli Studi del Piemonte Orientale, Via Solaroli 17, 28100 Novara, Italy; 2 Epidemiologic Unit, Vercelli Local Health Authority, Largo Giuseppe Giusti 13, 13100 Vercelli, Italy; 3 Department of Translational Medicine, Università del Piemonte Orientale, Via Solaroli 17, 28100 Novara, Italy; 4 Institute of Legal Medicine, Università “Magna Grecia” di Catanzaro, Catanzaro, Italy

**Keywords:** Healthy aging, Socioeconomic differences, Noncommunicable diseases, Community, Prevention, Health inequalities

## Abstract

Noncommunicable diseases (NCDs) are the leading global cause of death. The Italian National Prevention Plan (INPP) highlights the importance of health promotion and NCD prevention while avoiding health inequalities. In line with the INPP guidelines, we initiated a multicomponent community-based intervention program, named the Dedalo project, to promote healthy and active aging among population living around the Vercelli’s municipality, Italy. A cross-sectional analysis, that used the baseline data of a longitudinal study of the project, evaluated the program’s ability to enroll participants participants who represent the socioeconomic conditions present in the municipality. To this end, we compared the main social characteristics and behaviors of 40–74-year-old subjects (n = 155), who had attended at least one Dedalo activity, to those of same age individuals (n = 124) randomly extracted from the general population. We found that most participants were women (81.3%) and had a higher SES compared to the general population. Furthermore, they were healthier—OR 0.52, 95% CI 0.31–0.87 for self-reported diseases—and displayed healthier behaviors—OR 0.20, 95% CI 0.08–0.46 for smokers; 0.32 95%, CI 0.16–0.64 for fruit/vegetable consumers; and 0.36 95% CI 0.20–0.64 for sweet beverages consumers. Overall, our initial evaluation indicates that the Dedalo project has so far struggled to enroll individuals with low SES, men with any SES, and subjects displaying unhealthy behaviors, thereby failing to meet the INPP goal of preventing NCDs while avoiding health disparities. Thus, efforts should be made to ensure that this community-based intervention program can effectively reach all the target population, in particular those individuals most exposed to behavioral risk factors.

## Introduction

Noncommunicable diseases (NCDs) are defined by the World Health Organization (WHO) as "health problems that require continuous treatment over a period of time from years to decades" due to a combination of genetic, physiological, environmental, and behavioral factors (Gakidou et al., 2017). Currently, NCDs are the leading global cause of death (Skolnik, 2018), causing more deaths than all other causes combined, with over 77% of all NCD deaths occurring in low- and middle-income countries (WHO, 2011).

Among the six WHO regions, Europe is the most affected by NCDs, and the impact of the major NCDs—i.e., diabetes, cardiovascular diseases, cancer, chronic respiratory diseases, and mental disorders—is increasing at an alarming rate, accounting for an estimated 86% of deaths and 77% of disease burden [evaluated in Disability-Adjusted Life Years—DALY (Benziger et al., 2016)] (Gakidou et al., 2017).

In the pre-COVID-19 era, most deaths in Italy were caused by NCDs, with ischemic heart diseases, stroke, and Alzheimer occupying the first three positions in the ranking of death causes (Vos et al., 2020). In 2019, despite decreasing trends, the majority of risk factors were found to be mainly related to unhealthy behaviors: tobacco use, alcohol intake, high fasting plasma glucose, high blood pressure, overweight, diet (Murray et al., 2020).

The aging of the Western population is typically accompanied by increased NCD burden (Vos et al., 2020). According to the estimates of the Italian National Institute of Statistics (Istat), in 2019, 32.3% of the Italian elderly suffered from chronic diseases, 6.9% had impaired autonomy in their daily life (care and domestic tasks), significant enough to require caregivers, and 4.2% of them had dementia or Alzheimer’s disease (Istat, 2021). As the number of old age people will continue to increase for the foreseeable future, reaching an estimated 25% of the general population by 2027 (Istat, 2018), the prevalence of old age-associated NCDs and their ensuing financial burden is expected to grow exponentially (Istat, 2018).

In many high-income countries, such as Italy, NCDs are also a major cause of health disparities. The social determinants have in fact been shown to influence the distribution of the four main behavioral risk factors (unhealthy diet, physical inactivity, tobacco smoking, and excess alcohol consumption) (Marmot & Bell, 2019). Furthermore, individuals with high socioeconomic status (SES) are more likely to join prevention programs against NCDs (WHO, 2010), leading to a further increase in health disparities. In light of this, the Italian National Prevention Plan (INPP) advocates the design and implementation of evidence-based health promotion and preventive intervention programs capable of reducing health inequalities (Ministero della Salute—Direzione Generale della Prevenzione Sanitaria 2020).

In this scenario, the Italian PASSI surveillance system has revealed that, among individuals residing in the municipality of Vercelli, Italy (47,000 inhabitants), the 33% of sedentary people, the 45% with a BMI ≥ 25 (overweight/obese), and the 24% who declared to smoke regularly tobacco were in the age class 50–69 years (Epicentro - ISS, 2019a). Thus, in order to counteract a likely rise in the prevalence of NCDs, in 2019, the Local Health Authority (LHA) of Vercelli, together with University of Piemonte Orientale and the municipality of Vercelli, launched the Dedalo project, a community-based intervention program aimed to promote healthy and active aging through the development of cognitive and relational abilities. It takes place in the community setting involving and coordinating local agencies and stakeholders that can contribute to offering prevention interventions to citizens (ASL VC, 2021). An evaluation study was also putted in place in order to evaluate the impact on health of the project.

As INPP guidelines require that public health intervention for prevention and control of NCDs must ensure a concomitant reduction in health disparities, the aim of the study is to assess the Dedalo project’s ability to enroll participants whose socioeconomic status reflects that of the general population. The implications of our findings are discussed in light of future program improvements.

## Methods

### Study Design

We cross-sectionally analyzed data of the baseline of a prospective study initiated in March 2018 to evaluate the effectiveness of the Dedalo project in promoting healthy habits and behaviors among Italians aged 40–74 years.

The protocol of the study is registered at ClinicalTrial.Gov (clinical trial registration No. NCT03939754). The ethical approval was obtained from the Ethics Committee of Alessandria “Comitato Etico Interaziendale ASO SS. Antonio e Biagio e C. Arrigo” (No. AslVC.Med.18.02).

### Population, Enrollment, and Inclusion Criteria

All participants in the Dedalo project from March to December 2018, aged 40–74 years, were deemed eligible for this study. The study participants were enrolled during a Dedalo activity only after obtaining their written consent. Exclusion criteria were language other than Italian and the refusal to sign the informed consent form before enrollment. The control group was a random sample of individuals aged 40–74 extracted from the population registry of the municipality of Vercelli, stratified by age classes and sex. These subjects were enrolled through computer-aided telephone interview (CATI). Subjects who had participated in any Dedalo activity were deemed ineligible for the control group.

The target number of people to be enrolled in the study was 428 (214 for each group). This sample size was estimated considering the following data from literature: i) a 28–43% increase in prevalence of physically active adult population (data of Emp-H study—http://www.emp-h-project.eu/); ii) a 33% prevalence of inactive adult people living in Vercelli (Epicentro - ISS, 2019a); iii) an expected reduction of sedentary people by 35% after the intervention. The confidence intervals (CIs) and the power chosen were 95% and 80%, respectively. However, of the 345 participants in the Dedalo activities deemed eligible for the study, only 155 subjects completed the baseline interview. Thus, the total number of study participants was 369: 155 for the exposure group and 214 for the control group.

### Intervention

The Dedalo project was implemented thanks to a joint effort of the Vercelli’s LHA, the University of Piemonte Orientale (UPO), the municipality of Vercelli, and several other local organizations. It aims to promote healthy aging among the people residing in the municipality of Vercelli. The project takes place in the community setting developing community networks and mobilizing local stakeholders around a supply of “preventive opportunities” for the population (by networking building, coordination of actors and dissemination / communication of the Dedalo events to the population). To this end, the project has created a network of public and private entities, sports and cultural associations, volunteer associations, and other stakeholders to identify those activities that would best improve habits related to diet and physical activity in order to mitigate the risk of NCDs among the target population. The selected activities meeting the scientific standards of effectiveness are then offered to the community (Gottfredson et al., 2015).

Overall, the project is a multicomponent intervention characterized by social marketing and community coalition building organized in four paths: “Good diet, good health”, “Let’s work out together”, “Surprise and amazement”, and “Discovery of the territory”. The “good diet, good health” path, in Italian “Buona alimentazione”, is designed to provide information on how to adopt a healthy diet as a way to promote healthy aging. This is achieved through the organization of cooking courses, workshops, seminars, and thematic dinners. The "Let’s work out together" path, in Italian “Muoversi insieme”, fosters group exercising by making available to the participants gym classes, walking groups, and martial art courses. The "Surprise and amazement" path (in Italian “Meraviglia e stupore”) aims to promote socialization and cultural activity through museum visits, reading experiences, and active theatre and music experiences. The interventions envisaged by this path also seek to make healthcare settings (i.e., hospitals, nursing homes) more human through the introduction of cultural experiences (i.e., art exhibitions, conferences). Lastly, through the "Discovery of the territory" path (“Scoperta del territorio”), our project aims to promote both active life and the discovery of territory by organizing events such as hiking or walking in local forests and natural parks.

The teaching was carried out in the form of meeting and classes. A series of videos available on the Dedalo website (https://progettodedalo.net/, www.facebook.com/ProgettoDedaloVercelli/) were also shared with the participants.

### Questionnaire and Data Collection

The baseline survey of the longitudinal study was carried out from September to December 2018 using a standardized structured questionnaire. Data were collected by means of the REDCap software (version 9.5.14—© 2021 Vanderbilt University).

The questionnaire investigated the following aspects: (1) socio-demographic data (age, gender, education level, nationality, and the length of their stay in Italy, in the case of foreign nationalities), (2) perception of economic problems; (3) daily diet (fruit and vegetable, sweet drinks, and salt intake); (4) smoking habits and alcohol intake (daily drinking and binge drinking); (5) regular practice of physical exercises—by measuring their intensity levels, duration, and weekly frequency; (6) health status (diseases, medication intake and self-monitoring of blood pressure); (7) anthropometric parameters (height and weight); (8) satisfaction and wellbeing; (9) limitations of daily life due to physical and/or mental conditions; (10) active mobility (bicycle and walking); (11) active life at work; (12) attendance to cultural and social activities; and (13) knowledge of the Dedalo project and participation in its initiatives. The questionnaire (Annex 1) was based on validated questions extracted from the data collection tools of two national surveys, PASSI and Passi D’Argento (Epicentro - ISS, 2019a, 2019b).

In order to improve the response rate of the survey, the interviewers made as many as five call attempts for each candidate, two of which in the evening (after 7:00 pm). Only after five unsuccessful attempts or in case of refusal, the candidate was substituted. Subjects of the sample that were institutionalized (hospital, retirement home, prison) were also replaced with same age and gender candidates. The informed consent was collected at the beginning of each call. The data were stored in compliances with the data confidentiality and in accordance with the Italian legislation (Legislative Decree 196/2003) and the General Data Protection Regulation (EU) 2016/679 (GDPR).

### Measurements and Outcomes

Outcomes of the evaluation study were smoking and diet (fruit and vegetables, salt, and sugar drink intake), physical exercise (frequency and duration of moderate and high-level intensity), alcohol intake (daily intake and binge drinking), body mass index (BMI), cognitive activity, socialization, and wellbeing. For this cross-sectional analysis, the outcome was “participation to Dedalo project” and the above variables were studied as behavioral risk or protective factors and psychophysical conditions associated with chance to participate in Dedalo.

The following continuous variables were categorized: *i)* physical activity (< / ≥ 150 min of moderate physical activity/week *vs* 75 min of intense physical activity/week) (WHO, 2020); *ii)* fruit and vegetable daily intake (< / ≥ 5 portions/die); *iii)* BMI (< 25 kg/m^2^; 25–30 kg/m^2^; ≥ 30 kg/m^2^). Sugar drink consumption was measured with a relative frequency scale and analyzed considering two categories: never or < 1 glass of sugar drinks per week; and > 1 glass of sugar drinks per day.

About smoking and alcohol consumption, the first was classified into non-smoker, current smoker (i.e., smoker for more than 6 months before the interview day), and former smoker. Alcohol intake was also measured with a scale of relative frequency and was investigated with information about daily drinking and binge drinking, with this latter personalized by gender. This led to the following classification: no alcohol intake; 1 alcohol unit per day; 2–3 units per day; and 4 or more units per day.

Two classes encompassing cognitive and socialization activities were established: no active attendance in the past month, one or more listed attendance activities. Physical or psychological illnesses, days of physical inability, days of psychological inabilities were all classified according to the number of days reported: none, from 1 to 5, > 5 days in the last 30 days. The variable “Life satisfaction”, a single item scale with partial semantic autonomy of categories (very little, little, sufficient, highly), was classified in two other sub-groups: “medium-highly satisfied” and “poorly or not at all satisfied”.

Age was analyzed as both continuous and categorical variable, considering 5 age classes: < 50, 50–59, 60–69, and ≥ 70 years old. Three classes of education levels were defined: middle school or less; high school; and University degree. Satisfaction for economic resources was measured with a single item scale with partial semantic autonomy of categories, with four categories ranging from “very little difficulty” to “high difficulty”. This variable was then analyzed using two categories: presence or absence of reported economic difficulties. Finally, two classes of occupation status were defined: “worker” and “retired or other”.

### Statistical Analysis

Descriptive statistics of the whole study population or of the two separate groups (people exposed to the Dedalo’s interventions *vs* people not exposed) were carried out.

The categorical variables were reported as absolute number and percentage; the continuous variables were reported as mean, minimum and maximum value, and standard deviation (SD).

To test the differences for the categorical variables between the two groups, the Chi^2^ or Fisher test was performed as appropriate. In order to detect any differences between the continuous variables between the two groups, the normal distribution of the variables was assessed through the Shapiro–Wilk test; consequently, refusing the null hypothesis, the Mann Whitney nonparametric test was performed. The statistical significance was established for a p-value ≤ 0.05.

To test the association between the outcome variable “to be in the exposure group” and the studied variables, a univariate regression logistic model was performed. These analyses were described reporting the p-value, the odds ratio (OR) and the confidence interval at 95% (95% CI). Analysis was performed using as reference category the best condition for the participant respect to a healthy and active ageing (lack of risk factors / presence of protective factors). So, for example we analyzed the chance to participate to the Dedalo activities passing from the best conditions (daily intake of ≥ 5 fruit/vegetable portions) to the worsts (not any daily intake of fruits / vegetables). The multivariate regression logistic analysis was performed in order to adjust the association analyses for gender and age-class.

The statistical analyses were performed using the statistical software Stata MP 13.

## Results

**Fig. 1:**
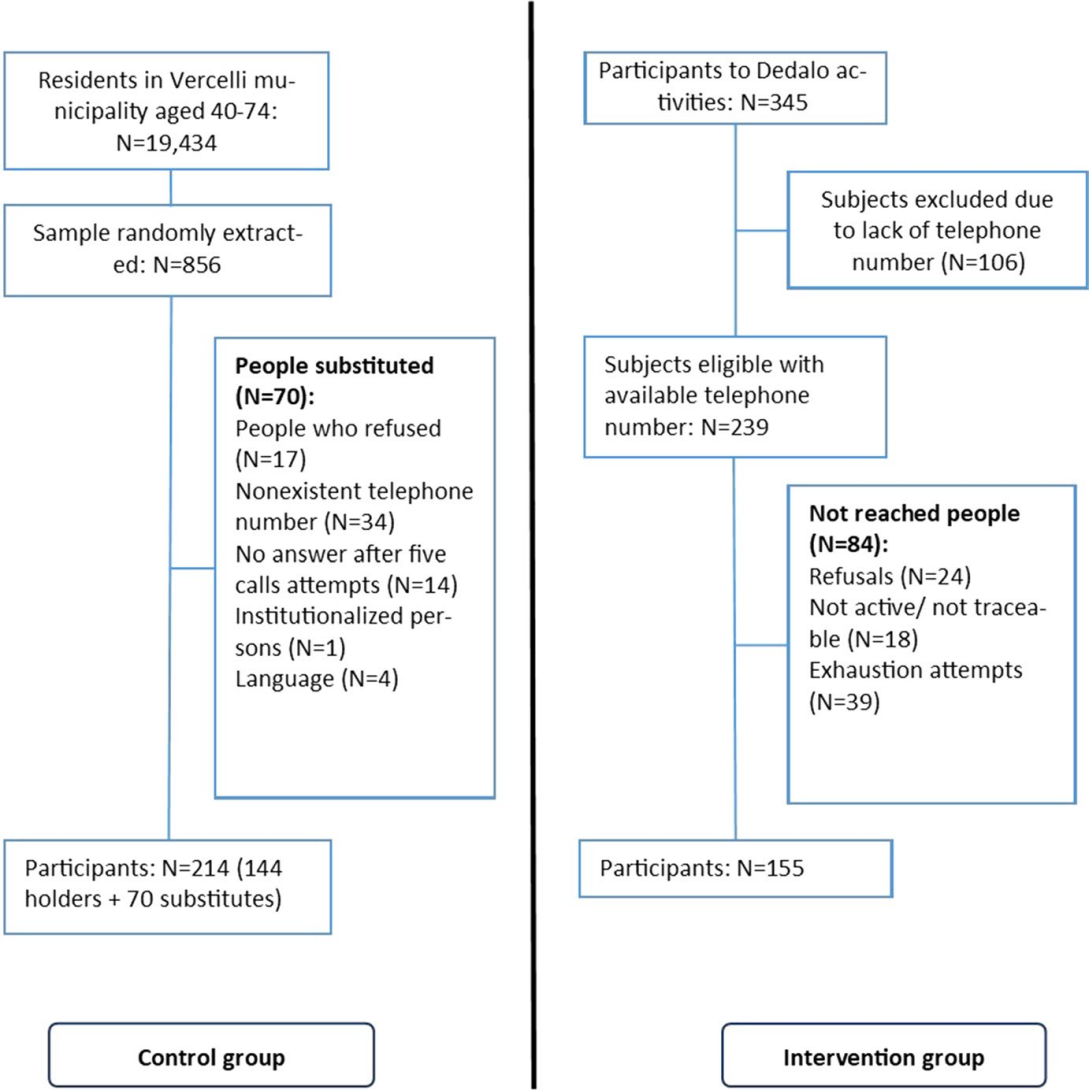
Flowchart of the study population showing the selection process of participants in the survey

People attending any of the Dedalo activities from March to December 2018 amounted to 345, of whom 239 provided a valid telephone number where they could be reached. Of them, 155 (64.9%) completed the baseline interview and were therefore enrolled in the study. The responders of the control group were 214, who came from a larger pool of 856 subjects residing in the municipality of Vercelli randomly extracted (Fig. 1).

The analyses of the demographic characteristics showed that the two groups under study did not present any statistically significant differences in the mean age (58.97 ± 8.84 vs. 57.59 ± 10.09 in Dedalo participants and control groups, respectively; p-value 0.1647). On the other hand, statistically significant differences emerged for distributions of the following variables in the two groups: age classes, sex, education level, and economic difficulties. Thus, among Dedalo participants 50–69 old-age women with higher educational levels and perceiving lower economic difficulties are more represented than in the control group. By contrast, no differences were found in occupational status (worker *vs* retired/other, p-value = 0.45) and citizenship (Italy vs. other countries, p-value = 0.48) (Table 1).

**Table 1:** Sociodemographic features of study participants about sex, age, education level, economic difficulties, and occupational status declared during the interviews

	Dedalo participants (%) (n = 155)	Control (%) (n = 214)	Total % (n = 369)	*p*-value
*Sex*				
Males	29 (18.7%)	104 (48.6%)	133 (36.3%)	< 0.001^b^
Females	126 (81.3%)	110 (51.4%)	236 (64.0%)	
*Age classes, years*				
< 50	27 (17.4%)	58 (27.1%)	85 (23.0%)	< 0.001^a^
50–59	48 (31.0%)	60 (28.0%)	108 (29.3%)	
60–69	63 (40.7%)	51 (23.8%)	114 (30.9%)	
≥ 70	17 (11.0%)	45 (21.0%)	62 (16.8%)	
*Education level*				
None/ primary or middle school	23 (14.8%)	66 (30.8%)	89 (24.1%)	< 0.001^a^
High school	82 (52.9%)	116 (54.2%)	198 (53.7%)	
University degree	50 (32.3%)	32 (15.0%)	82 (22.2%)	
*Economic difficulties*^*c*^				
Yes	41 (26.6%)	84 (39.2%)	125 (34.0%)	0.012 ^b^
No	113 (73.4%)	130 (60.8%)	243 (66.0%)	
*Occupational status*^*d*^				
Workers	56 (48.7%)	76 (53.9%)	132 (51.6%)	0.451 ^b^
Retired and other non-workers	59 (51.3%)	65 (46.1%)	124 (48.4%)	
*Citizenship*				
Italy	152 (98.7%)	208 (97.2%)	360 (97.8%)	0.477 ^b^
Other countries	2 (1.3%)	6 (2.8%)	8 (2.2%)	

^a^*p*-value of Chi-2 test

^b^*p*-value of Fisher’s exact test

^c^For this variable, 1 missing data

^d^For this variable, 113 missing data, 40 and 73 in exposure and unexposure groups, respectively

Table 2 shows distribution between the two groups of the behavioral risks. Differences between groups were statistically significant for all variables except for BMI. Dedalo participants had self-report higher weekly minutes of moderate physical activity (by contrast, control group declared higher weekly minutes of intense physical activity), major attention in salt intake, none sugar drink consumption, less daily alcohol consumption, and more social activities participation in comparison to the control. In addition, the exposure group had self-report most often never smoke habits respect to the unexposure one (65.6% vs. 46.2%, respectively) (see Table 2).

**Table 2:** Behavioral risk factors distribution among exposure and unexposure groups

Risk factors	Dedalo participants (%) (n = 155)	Controls (%) (n = 214)	Total (%) (n = 369)	*p*-value
*BMI*				
< 25	92 (59.4%)	106 (49.5%)	198 (53.7%)	0.157^a^
25 – 29	47 (30.3%)	77 (36.0%)	124 (33.6%)	
≥ 30	16 (10.3%)	31 (14.5%)	47 (12.7%)	
*Fruit/vegetable portions*				
≥ 5	33 (21.3%)	17 (7.9%)	50 (13.5%)	< 0.001^b^
< 5	122 (78.7%)	197 (92.1%)	319 (86.5%)	
*Smoke*				
Never	101 (65.6%)	99 (46.2%)	200 (54.4%)	< 0.001^a^
Ex-smoker	45 (29.2%)	59 (27.6%)	104 (28.3%)	
Current smoker	8 (5.2%)	56 (26.2%)	64 (17.4%)	
*Minutes/week of moderate physical activity*				
≥ 150	59 (40.0%)	62 (25.6%)	121 (32.8%)	0.014^b^
< 150	155 (60.0%)	93 (72.4%)	248 (67.2%)	
*Minutes/week of intense physical activity*				
≥ 75	14 (9.0%)	35 (16.4%)	49 (13.3%)	0.044^b^
< 75	141 (91.0%)	179 (83.6%)	320 (86.7%)	
*Salt intake attention*				
Yes	129 (83.2%)	155 (72.4%)	284 (77.0%)	0.017^b^
No	26 (16.8%)	59 (27.6%)	85 (23.0%)	
*Consumption of sugar drinks, glasses/week*				
Never	133 (85.8%)	137 (64.0%)	270 (73.2%)	< 0.001^b^
1 or more	22 (14.2%)	77 (36.0%)	99 (26.8%)	
*One or more cognitive/socialization activities in the last 30 days*				
Yes	149 (96.1%)	189 (88.3%)	338 (91.6%)	0.007^b^
No	6 (3.9%)	25 (11.7%)	31 (8.4%)	
*Alcohol intake, glasses/day*				
Never	35 (22.9%)	66 (30.8%)	101 (27.5%)	0.042^a^
≤ 1	103 (67.3%)	125 (58.4%)	228 (62.1%)	
2–3	12 (7.8%)	23 (10.8%)	35 (9.5%)	
4 or more	3 (2.0%)	0 (0.0%)	3 (0.8%)	

^a^*p*-value of Chi-2 test

^b^*p*-value of Fisher’s exact test

Psychophysical conditions are showed in Table 3. Differences between groups emerged for *Unhealthy days* for psychological conditions, life satisfaction, diagnosis of myocardial infarction, cancer, and arthrosis. Thus, in comparison to control group, Dedalo participants self-reported most often a medium–high level of life satisfaction, a higher number of unhealthy days for psychological conditions, less diagnosis of myocardial infarction, arthrosis but more diagnosis of cancer (see Table 3).

**Table 3:** Psychophysical conditions distribution among exposure and unexposure groups

Psychophysical conditions	Dedalo participants % (N = 155)	Controls % (N = 214)	Tot. % (N = 369)	*p*-value
*Life satisfaction *^*c*^				
Medium–high	144 (93.5%)	169 (79.3%)	313 (85.3%)	< 0.001^b^
Low	10 (6.5%)	44 (20.7%)	54 (14.7%)	
*Physical illness, No. of unhealthy days in the last month*				
0	96 (61.9%)	133 (62.2%)	229 (62.1%)	0.459^a^
≤ 5	34 (21.9%)	55 (25.7%)	89 (24.1%)	
> 5	25 (16.2%)	26 (12.2%)	51 (13.8%)	
*Psychological conditions, No. of unhealthy days in the last month*				
0	77 (49.7%)	148 (69.2%)	225 (61.0%)	< 0.001^a^
≤ 5	39 (25.2%)	26 (12.2%)	65 (17.6%)	
> 5	39 (25.2%)	40 (18.7%)	79 (21.4%)	
*Self-reported diseases at least one*				
No	68 (43.9%)	80 (37.4%)	148 (40.1%)	0.237^b^
Yes	87 (56.1%)	134 (62.6%)	221 (59.9%)	
*Self-reported diseases*^*b*^				
Diabetes	6 (3.9%)	11 (5.1%)	17 (4.6%)	0.624
Respiratory illness	6 (3.9%)	15 (7.0%)	21 (5.7%)	0.257
Hypertension	45 (29.0%)	70 (32.7%)	115 (31.2%)	0.495
Myocardial infarction	2 (1.3%)	13 (6.1%)	15 (4.1%)	0.030
Other heart diseases	14 (9.0%)	9 (4.2%)	23 (6.2%)	0.080
Ictus	2 (1.3%)	4 (1.9%)	6 (1.6%)	1.000
Liver diseases	3 (1.9%)	4 (1.9%)	7 (1.9%)	1.000
Cancer	26 (16.8%)	19 (8.9%)	45 (12.2%)	0.025
Arthrosis	36 (23.2%)	77 (36.0%)	113 (30.6%)	0.009
*Treated hypertension*				
No	115 (74.2%)	145 (67.8%)	260 (70.5%)	0.204^b^
Yes	40 (25.8%)	69 (32.2%)	109 (29.5%)	

^a^*p*-value of Chi-2 test

^b^*p*-value for Fisher’s exact test

^c^For this variable, 2 missing data

Table 4 shows the chance to participate to the Dedalo’s activities passing from the best conditions, that is charactered by lack of risk factors / presence of protective factors (i.e., daily intake of ≥ 5 fruit/vegetable portions, BMI < 25, etc.), to the worsts (i.e., not any daily intake of fruits and vegetables, BMI ≥ 30, etc.). Participants in the Dedalo project were less exposed to all the analyzed risk factors. In the model adjusted by sex and age class, they were significantly less exposed to a low consumption of fruits and vegetables, to current smoking (OR = 0.20, CI 95% 0.08–0.46), and consumption of sugar drinks. No association emerged for alcohol consumption and physical activity in the adjusted models.

**Table 4:** Risk factor distribution for exposure and unexposure groups analyzed in the evaluation study of the Dedalo project: unadjusted and adjusted (by sex and age classes) logistic models for the chance to participate to Dedalo

Risk factors	Dedalo participants % (n = 155)	Controls % (n = 214)	OR unadjusted, CI 95%	OR adjusted^a^, CI 95%
*BMI*				
< 25	92 (59.4%)	106 (49.5%)	1	1
25—29	47 (30.3%)	77 (36.0%)	0.70 (0.45–1.11)	1.00 (0.59–1.68)
≥ 30	16 (10.3%)	31 (14.5%)	0.60 (0.31–1.16)	0.81 (0.38–1.72)
*Fruit/vegetable portions*				
≥ 5	33 (21.3%)	17 (7.9%)	1	1
< 5	122 (78.7%)	197 (92.1%)	**0.32 (0.17–0.60)**	**0.32 (0.16–0.64)**
*Smoke*				
Never	101 (65.6%)	99 (46.2%)	1	1
Ex-smoker	45 (29.2%)	59 (27.6%)	0.75 (0.46–1.20)	1.20 (0.67–2.13)
Current smoker	8 (5.2%)	56 (26.2%)	**0.14 (0.06–0.31)**	**0.20 (0.08–0.46)**
*Minutes/week of moderate physical activity*				
≥ 150	59 (40.0%)	62 (25.6%)	1	1
< 150	155 (60.0%)	93 (72.4%)	**0.57 (0.37–0.89)**	0.77 (0.47–1.26)
*Minutes/week of intense physical activity*				
≥ 75	14 (9.0%)	35 (16.4%)	1	1
< 75	141 (91.0%)	179 (83.6%)	1.97 (1.02, 3.80)	1.53 (0.72–3.23)
*Salt intake attention*				
Yes	129 (83.2%)	155 (72.4%)	1	1
No	26 (16.8%)	59 (27.6%)	**0.53 (0.32–0.89)**	0.63 (0.35–1.12)
*Consumption of sugar drinks, glasses/week*				
0	133 (85.8%)	137 (64.0%)	1	1
1 or more	22 (14.2%)	77 (36.0%)	**0.29 (0.17–0.50)**	**0.36 (0.20–0.64)**
*One or more cognitive/socialization activities in the last 30 days*				
Yes	149 (96.1%)	189 (88.3%)	1	1
No	6 (3.9%)	25 (11.7%)	**0.30 (0.12–0.76)**	**0.40 (0.15–1.07)**
*Alcohol intake, glasses/day*				
Never	35 (22.9%)	66 (30.8%)	1	1
≤ 1	103 (67.3%)	125 (58.4%)	1.55 (0.96–2.53)	1.60 (0.92–2.79)
2–3	12 (7.8%)	23 (10.8%)	0.98 (0.44–2.21)	1.61 (0.62–4.19)
4 or more	3 (2.0%)	0 (0.0%)	–	–

95% CI are statistically significant for lower fruits and vegetables intake (<5 daily portions), smoking habits (current smokers), sugar intake (one or more sugar drink glasses per week), socialization habits (no socialization activities in the last 30 days)

*CI * confidence interval, *OR* odds ratio

^a^Models were adjusted for sex and age classes

Considering the inferential analysis results, the participants in the Dedalo project reported a lower chance of referring heart attacks and arthrosis or a treatment for hypertension, but higher risk of have diagnosis heart diseases (Table 5). Considering quality of life, no differences emerged in unhealthy days for physical illness, whereas Dedalo participants showed a worst psychological status with 88% of chance higher than control to had experience at least 5 unhealthy days for mental distress in the last month. Thus, even though the unexposure group resulted have higher self-reported psychological wellness compared to exposure one, the percentage of individuals reporting high levels of “life satisfaction” was higher in Dedalo group than the control: passing from the best condition (medium–high level of life satisfaction) to the worst (low level) the chance to not attendance to Dedalo activities increase (73% higher compared to control in the both adjusted and unadjusted models, Table 5). Furthermore, the exposure individuals were generally healthier, with 43.9% of them not reporting any of the investigated diseases *vs* 37.4% of controls.

**Table 5:** Psychophysical conditions of the population observed in the evaluation study of the Dedalo project: unadjusted and adjusted (by sex and age classes) logistic models for the chance to participate to Dedalo

Psychophysical conditions	Dedalo participants % (N = 155)	Controls % (N = 214)	OR unadjusted, CI 95%	OR adjusted^a^, CI 95%
*Life satisfaction*^*b*^				
Medium–high	144 (93.5%)	169 (79.3%)	1	1
Low	10 (6.5%)	44 (20.7%)	**0.27 (0.13–0.55)**	**0.27 (0.12–0.60)**
*Physical illness, No. of unhealthy days in the last month*				
0	96 (61.9%)	133 (62.2%)	1	1
≤ 5	34 (21.9%)	55 (25.7%)	0.86 (0.52–1.41)	0.69 (0.39–1.24)
> 5	25 (16.2%)	26 (12.2%)	1.33 (0.73–2.45)	1.11 (0.55–2.25)
*Psychological conditions,* *No. of unhealthy days in the last month*				
0	77 (49.7%)	148 (69.2%)	1	1
≤ 5	39 (25.2%)	26 (12.2%)	**2.88 (1.63–5.09)**	**1.88 (1.01–3.51)**
> 5	39 (25.2%)	40 (18.7%)	**1.87 (1.11–3.15)**	1.40 (0.78–2.54)
*Self-reported diseases*				
Diabetes	6 (3.9%)	11 (5.1%)	0.74 (0.27–2.06)	0.99 (0.32–3.01)
Respiratory illness	6 (3.9%)	15 (7.0%)	0.53 (0.20–1.41)	0.73 (0.25–2.16)
Hypertension	45 (29.0%)	70 (32.7%)	0.84 (0.54–1.32)	0.67 (0.40–1.12)
Myocardial infarction	2 (1.3%)	13 (6.1%)	**0.20 (0.05–0.91)**	**0.17 (0.03–0.88)**
Other heart diseases	14 (9.0%)	9 (4.2%)	2.26 (0.95–5.37)	**3.17 (1.15–8.69)**
Ictus	2 (1.3%)	4 (1.9%)	0.69 (0.12–3.80)	0.42 (0.07–2.67)
Liver diseases	3 (1.9%)	4 (1.9%)	1.04 (0.23–4.70)	1.78 (0.30–10.41)
Cancer	26 (16.8%)	19 (8.9%)	2.07 (1.10–3.89)	1.78 (0.86–3.69)
Arthrosis	36 (23.2%)	77 (36.0%)	**0.54 (0.34–0.86)**	**0.32 (0.18–0.57)**
*Self-reported diseases at least one*				
No	68 (43.9%)	80 (37.4%)	1	1
Yes	87 (56.1%)	134 (62.6%)	0.75 (0.50–1.16)	**0.52 (0.31–0.87)**
*Treated hypertension*				
No	115 (74.2%)	145 (67.8%)	1	1
Yes	40 (25.8%)	69 (32.2%)	0.73 (0.46, 1.16)	**0.59 (0.34, 1.00)**

95% CI are statistically significant for lower life satisfaction, few unhealthy days in the last month for psychological conditions, people affected by myocardial infarction, other heart diseases, and arthrosis, self-reported at least a diagnosticated disease, people declared drug intake for hypertension

*Tot* total, *CI* confidence interval, *OR* odds ratio

^a^Models were adjusted for sex and age classes

^b^For this variable, 2 missing data

Finally, Table 6 presents results of an adjusted (by sex and age) multivariate logistic regression including behavioral risk factors (smoke habits, sugar drinks consumption) and psychophysical conditions (life satisfaction, unhealthy days for mental distress, diagnosis of myocardial infarction, and heart disease) associated to the outcome in the adjusted models by sex and age (Tables 4 and 5).

**Table 6:** Behavioral risk factors and psychophysical conditions of the population observed in the evaluation study of the Dedalo project: adjusted (by sex and age classes) logistic multivariate model for the chance to participate to Dedalo

	Dedalo participants % (N = 155)	Controls % (N = 214)	OR adjusted^a^, CI 95%
*Smoke*			
Never	101 (65.6%)	99 (46.2%)	1
Ex-smoker	45 (29.2%)	59 (27.6%)	1.13 (0.63–2.07)
Current smoker	8 (5.2%)	56 (26.2%)	**0.19 (0.08–0.45)**
*Consumption of sugar drinks, glasses/week*			
0	133 (85.8%)	137 (64.0%)	1
1 or more	22 (14.2%)	77 (36.0%)	**0.41 (0.22–0.75)**
*Life satisfaction*^*b*^			
Medium–high	144 (93.5%)	169 (79.3%)	1
Low	10 (6.5%)	44 (20.7%)	**0.22 (0.09–0.51)**
*Psychological conditions,* *No. of unhealthy days in the last month*			
0	77 (49.7%)	148 (69.2%)	1
≤ 5	39 (25.2%)	26 (12.2%)	**2.59 (1.34–5.00)**
> 5	39 (25.2%)	40 (18.7%)	**2.01 (1.02–3.97)**
*Self-reported diseases*			
Myocardial infarction	2 (1.3%)	13 (6.1%)	**0.18 (0.03–0.94)**
Other heart diseases	14 (9.0%)	9 (4.2%)	1.79 (0.61–5.28)

95% CI are statistically significant for smoking habits (current smokers), sugar intake (one or more sugar drink glasses per week), lower life satisfaction, number of unhealthy days in the last month for psychological conditions, self-reported diseases (myocardial infarction)

*CI * confidence interval, *OR* odds ratio

^a^Model was adjusted for sex and age classes and include the following variables, with low collinearity: smoke habits, sugar drinks consumption, life satisfaction, unhealthy days for mental distress, diagnosticated myocardial infarction and heart diseases

^b^ Life satisfaction was measured with a 5-points frequency item from "highly" to "very little" (very little, little, sufficient, highly). The first two points were categorized in low satisfaction, whereas the last two in medium-high satiscation

Thus, all associations were confirmed except for self-reported diagnosis of “Other heart diseases” and ex-smokers. Among factors associated to participation, we can note a considerable increase in ORs related to unhealthy days for mental distress (ORs 2.59 95% CI 1.34–5.00 and 2.01 95% CI 1.02–3.97 for ≤ 5 and > 5 unhealthy days for mental distress, respectively) (Table 6).

## Discussion

The purpose of this study is to assess the ability of the Dedalo project, a multicomponent community intervention for the promotion of healthy aging, to enroll the target population avoiding selection in terms of SES. This was done by using the baseline data of the longitudinal study aimed to evaluate the impact of the intervention on the main outcomes of the intervention. The project, implemented in Vercelli, a medium-small city in the North of Italy, consists of different types of interventions encompassing the following four themes: healthy diet, physical exercise, socialization and culture, and discovery of the local territory.

Our results suggest that the activities promoted by the Dedalo project are predominantly attended by women (81.3%) with a high education level and low economic difficulties, compared to unexposure controls randomly selected from the general population. The participants also tend to have healthier behaviors compared to the general population: they smoke rarely, consume a large amount of fruit and vegetables, and have a low intake of sugar drinks.

Another interesting observation is that people involved in the Dedalo’s interventions tend to be healthier than control subjects, except for the variable “other hearth diseases”. These characteristics are consistent with those observed in volunteers taking part in preventive interventions and health promotion activities within the community (Chylińska et al., 2017; Marmot & Bell, 2019; WHO, 2010). This is particularly true in Italy, where social behaviors, such as participation in community services, are inversely associated with education levels, economic difficulties, and age (Epicentro - ISS, 2019b). Our data are also in good agreement with previously published studies showing social inequalities in health (Marmot & Bell, 2019; WHO, 2010).

Dedalo project was also characterized by overwhelming attendance of women to its activities. This is not surprising given that older women in Italy are typically more engaged in social activities requiring voluntary work (elderly people as “resource” for family, community, and economy (WHO, 2002))—e.g., family or community caregiving initiatives—than men (32.4% of women vs. 23.7% of men), who instead participate more than women (23% and 21%, respectively) to community and cultural activities and events—e.g., community life, travels, and training activities (Epicentro - ISS, 2019b).

A last observation is worth to be done: the percentage of smokers in the population adhering to the project is one fifth of that of the control group and of the general Italian adult population (Epicentro - ISS, 2019a). Considering that people from lower SES groups are more likely to smoke and less likely to quit smoking than those from higher SES groups (Brown et al., 2014), our results appear to indicate a severe selection in the enrollment of the target population.

Altogether, the previous observations show that Dedalo project attracted a selected population, characterized to be of higher social class, to have a lower level of exposure to risk factors, and a better health and wellbeing, predominantly female.

The findings herein reported show that the Dedalo project has so far failed to reach one of its primary objectives: the reduction of social inequalities in health. Indeed, the observation that the vast majority of unhealthy behaviors were less frequent among the program participants compared to a random sample of the general population suggests that the individuals enrolled in the initiative were probably those in less need of preventive interventions. Assuming that socioeconomic status of the local population is distributed like the mean of the population of the Northern Italy, we hypothesize for Vercelli’s municipality that among people with 40 years old or more, about 45% have low educational levels and about the 7% (≥ 35 years old) are in relative poverty (Istat 2020a, 2020b). Considering the amount of population at risk (for SES), it is imperative that we identify alternative enrollment strategies capable of targeting people with lower SES and adult men, who at the moment do not seem to be particularly attracted by the program.

One possible explanation for this failure is that the communication channels that were used to advertise the various initiatives (e.g., fliers, newspaper ads, websites, social networks, and word of mouth) may have caused a selection bias, excluding the people most in need (i.e., lower SES subjects and adult men).

In this regard, a review on social marketing for behavior change has underscored several tools with which to achieve a more effective advertising for these types of initiatives (McKenzie-Mohr & Schultz, 2014). For instance, the promotion of healthy behaviors based on local social norms focusing on the social identity of the community or the use of a renowned influencer has proven highly effective in increasing the motivation levels of the target population to participate in an intervention.

The results of this study have been used to change the communication approach of the Dedalo activities, including more local channels in order to reach people in their neighborhoods and involving local associations in the conduction of the intervention. Furthermore, although Dedalo is a program for general population, its events and activities can be addressed also to a specific target population that could have great benefits with participation. For example, a walking group in Dedalo was planned for patients of Addiction Service, an “emotion artistic workshop” for oncologic patients, or dance therapy course for patients with Parkinson disease.

This cross-sectional analysis has a limit in the method used in the sampling. First, the type of variables used as *proxy* of SES indicator: educational level and economic difficulties perceived by participants. This last variable is very easy to measure with low risk of bias for *social desirability*, but is strongly subjective. So, to consider social inequality in the preventive intervention access, it would be recommended to use more objective indicators to measure SES status. Lastly, for the type of sampling stratification (by age and sex) of the randomized control, that likely have led to a low presence of people with foreign origin among unexposure group (about 3% of unexposure participants vs. about 7% of the total Vercelli’s residents with 40 years and more) (Istat, 2019).

Another possible limit is that the Dedalo activities are carried out in Italian, with the risk to exclude foreign citizens. The percentage of foreign people living in Vercelli is quite low and about 6,7% of the total residents (in the age class > 40 years, in 2019); moreover national data showed that in Italy about 64% of the foreign people have not any difficulty to understand Italian (Istat, 2019). Thus, this possible bias seems to have a low impact.

## Conclusion

This study is one of the first evaluations of a community intervention in Italy and, to our knowledge, in Europe. Our findings indicate that the Dedalo project has so far been unsuccessful in engaging in its initiatives the most vulnerable population to NCDs, thereby failing to meet the goal of reducing health disparities.

Even though our results are essentially negative, we strongly believe that they will provide a useful source of information to better understand the impact of preventive interventions on health inequalities within a community, thereby fostering future collaborative research endeavors.

## Data Availability

Due to the sensitive nature of the questions asked in this study, survey, respondents were assured that the raw data would remain confidential and would not be shared with third parties.

## References

[CR1] ASL VC (2021) Dedalo—Volare sugli anni. http://www.aslvc.piemonte.it/index.php?option=com_content&view=article&id=864:dedalo-volare-sugli-anni&catid=63:ambito-della-prevenzione. Accessed 14 Oct 2021.

[CR2] Benziger CP, Roth GA, Moran AE (2016). The Global Burden of Disease Study and the Preventable Burden of NCD. Global Heart.

[CR3] Brown T, Platt S, Amos A (2014). Equity impact of European individual-level smoking cessation interventions to reduce smoking in adults: A systematic review. European Journal of Public Health.

[CR4] Chylińska J, Łazarewicz M, Rzadkiewicz M (2017). The role of gender in the active attitude toward treatment and health among older patients in primary health care-self-assessed health status and sociodemographic factors as moderators. BMC Geriatrics.

[CR5] Epicentro—ISS (2019a). Sorveglianza PASSI. https://www.epicentro.iss.it/passi/dati/attivita?tab-container-1=tab1#nazionali. Accessed 20 Jul 2021.

[CR6] Epicentro—ISS (2019b). Sorveglianza Passi d’Argento. https://www.epicentro.iss.it/passi-argento/. Accessed 20 Jul 2021.

[CR7] Gakidou E, Afshin A, Abajobir AA (2017). Global, regional, and national comparative risk assessment of 84 behavioural, environmental and occupational, and metabolic risks or clusters of risks, 1990–2016: A systematic analysis for the Global Burden of Disease Study 2016. Lancet.

[CR8] Gottfredson DC, Cook TD, Gardner FEM (2015). Standards of evidence for efficacy, effectiveness, and scale-up research in prevention science: Next Generation. Prevention Science.

[CR9] Istat. (2021). Le condizioni di salute della popolazione anziana in Italia. Anno 2019. Roma.

[CR10] Istat (2018) Indicatori demografici. Stime per l’anno 2017. 1–15

[CR11] Istat. (2020a). I.Stat. In: Pop. e Fam. https://dati.istat.it. Accessed 26 Jan 2021.

[CR12] Istat. (2020b). I.Stat. In: Condizioni Econ. delle Fam. e disuguaglianze. http://dati.istat.it. Accessed 4 May 2022.

[CR13] Istat. (2019). I.stat. In: Demogr. cifre. https://demo.istat.it/. Accessed 6 May 2022.

[CR14] Marmot M, Bell R (2019). Social determinants and non-communicable diseases: Time for integrated action. BMJ.

[CR15] McKenzie-Mohr D, Schultz PW (2014). Choosing effective behavior change tools. Soc Mar Q.

[CR16] Ministero della Salute—Direzione Generale della Prevenzione Sanitaria. (2020). Piano Nazionale della Prevenzione 2020–2025. Roma.

[CR17] Murray CJL, Aravkin AY, Zheng P (2020). Global burden of 87 risk factors in 204 countries and territories, 1990–2019: A systematic analysis for the Global Burden of Disease Study 2019. Lancet.

[CR18] Skolnik, R. (2018). Noncommunicable Diseases Country Profiles 2018.

[CR19] Vos T, Lim SS, Abbafati C (2020). Global burden of 369 diseases and injuries in 204 countries and territories, 1990–2019: A systematic analysis for the Global Burden of Disease Study 2019. Lancet.

[CR20] WHO. (2011). Global status report on noncommunicable diseases 2010. Geneva.

[CR21] WHO. (2010). Western Pacific Region. Noncommunicable disease risk factors and socioeconomic inequalities—what are the links? A multicountry analysis of noncommunicable disease surveillance data Report to the WHO Regional Office for the Western Pacific Section.

[CR22] WHO. (2020). WHO Guidelines on physical activity and sedentary behaviour. Geneva.

[CR23] WHO. (2002). Active ageing. A policy framework. Geneva.

